# WHO International Health Regulations Emergency Committee for the COVID-19 outbreak

**DOI:** 10.4178/epih.e2020013

**Published:** 2020-03-19

**Authors:** Youngmee Jee

**Affiliations:** 1 Member of the WHO IHR Emergency Committee for COVID-19; 2 Global Center for Infectious Diseases, Seoul National University College of Medicine, Seoul, Korea

**Keywords:** COVID-19, International Health Regulations, Emergency Committee, Public Health Emergency of International Concern, Research and development

## Abstract

To discuss whether the coronavirus disease 2019 (COVID-19) outbreak constitutes a Public Health Emergency of International Concern (PHEIC), World Health Organization (WHO) organized the 15-member International Health Regulations Emergency Committee (EC). On January 22-23 and January 30, 2020, EC convened and discussed whether the situation in China and other countries would constitute PHEIC and issued recommendations for WHO, China and the international community. Based on the recommendations of EC, WHO declared the COVID-19 outbreak a PHEIC. One of the purposes of the declaration of PHEIC was to alarm countries with weak public health infrastructures to prepare promptly for emerging infectious diseases (EID) and provide WHO with a framework for proactively supporting those countries. On February 3, 2020, WHO proposed the 2019 COVID-19 Strategic Preparedness and Response Plan, which includes accelerating research and development (R&D) processes as one of three major strategies. On February 11-12, 2020, WHO held the Global Research and Innovation Forum: Towards a Research Roadmap for COVID-19. The fact that a COVID-19 R&D forum was the first meeting convened after the PHEIC declaration testifies to the importance of R&D in response to EID. Korea has demonstrated a remarkable capacity in its laboratory response by conducting high-throughput COVID-19 testing and utilizing innovative drive-through samplings. These measures for early detection and screening of cases should be followed by full efforts to produce research-based evidence by thoroughly analyzing epidemiological, clinical and immunological data, which will facilitate the development of vaccines and therapeutics for COVID-19. It is expected that Korea plays a global partner for COVID-19 research by actively participating in immediate and mid/long-term priorities jointly led by WHO and global partners.

The first and second teleconferences of the World Health Organization (WHO) International Health Regulations (IHR) Emergency Committee (EC) to discuss the situation of coronavirus disease 2019 (COVID-19) outbreak and whether WHO should declare a “Public Health Emergency of International Concern (PHEIC)” were held on January 22-23 and January 30, 2020 [1]. In the early morning of January 31, 2020 (Korea Standard Time), WHO declared the COVID-19 outbreak a PHEIC as stipulated by Article 12 of the IHR. To date, there have been six occasions of declaring PHEICs by WHO including the current COVID-19 outbreak. Previously declared cases include the 2009 novel flu pandemic, 2014 wild polio, 2014 West African Ebola, 2016 Zika virus, and 2018 Ebola outbreak.

Under the IHR revised in 2005, each country must report any event that may constitute a PHEIC to WHO within 24 hours. Based on the risk of international spread, and travel or trade restrictions during the public health emergency in the country, WHO establishes an EC to discuss whether PHEIC must be declared, and the final declaration is made by the WHO Director-General. For WHO to declare a PHEIC, the necessary criteria provided in Annex 2 of the IHR must be fulfilled [1]. This includes the assessment of significant risk of international spread, and impact of the infectious disease on travel or trade. Given that many cases of infectious disease occur during an outbreak and are reported to WHO every year, a PHEIC is determined by WHO after carefully assessing whether the reported event (1) has a serious public health impact, (2) is unusual or unexpected, (3) has significant risk of international spread, and (4) has significant risk of international travel or trade restrictions [1].

The current IHR COVID-19 EC consists of 15 experts, from various fields, representing the six WHO regions. The experts are from Australia, Canada, China, France, Japan, Netherlands, Russia, Saudi Arabia, Senegal, Singapore (2), Korea, Thailand (2) and United States. Their areas of expertise include epidemiology, virology, infectious diseases and public health as well as risk communications. Since PHEIC declaration requires a consensus of committee members, COVID-19 was not declared as a PHEIC at the first meeting held on January 22-23, 2020 as a consensus was not reached. After monitoring the development of community transmission in other countries, WHO formed a consensus in the second meeting held on January 30, 2020. The EC acknowledged that confirmed cases were reported in five WHO regions in one month, and human-to-human transmission occurred outside China. The EC also mentioned in a statement that interruption of virus spread is still possible if countries put strong measures to detect cases early, isolate and treat cases, trace contact and promote social distancing. It also noted that strategic goals and measures to prevent and reduce spread of infection could be revised in view of evolving situations.

Based on the advice of the EC, information provided by the affected countries, and data on the risk of international spread and problem of travel or trade restrictions, the WHO Director-General has the authority to make the final declaration of PHEIC.

The following is a summary of the statement of the EC, advising the Director-General Ghebreyesus on the determination of PHEIC (the three-point summary provided below is based on my editorial published in the *Hankook Ilbo* on February 3, 2020 [2]).

First, WHO shall send experts to China and organize a joint mission to address the situation. This joint mission team shall be similar to the joint assessment team organized during the Middle East Respiratory Syndrome outbreak in Korea in 2015. The difference is that in Korea, the joint mission was conducted first, resulting in no PHEIC declaration, while in China, emergency was declared first, and then the joint mission was conducted. The joint mission shall assess the situation of the COVID-19 outbreak and response measures taken in China, and will provide recommendations accordingly. In addition, it was emphasized that WHO shall provide support to countries and regions with vulnerable public health infrastructure and collaborate with them.

Second, the measures to be taken by China were listed. This included cooperation with WHO and related agencies and identification of infected travelers by conducting exit screening at airports and ports. In particular, a thorough exit screening for China was emphasized to minimize travel restrictions from China to other countries.

Third, the statement included that WHO shall not immediately recommend any travel or trade restrictions based on current information available. It was proposed that if a State Party was required to implement measures that constrained international traffic of travelers and goods, it must notify WHO of the public health justification within 48 hours for WHO to review the justification for such a measure. Moreover, the State Parties were cautioned not to promote stigma or discrimination against specific groups, in accordance with the human rights principles of Article 3 of the IHR.

The primary aim of WHO, upon declaration of PHEIC, is to prevent the international spread of the infectious disease as much as possible and support the response of the affected country, while avoiding imposing excessive trade restrictions. IHR states that the following measures shall be taken when WHO declares a PHEIC (Article 13 Public Health Response):

First, each State Party shall secure the capacity to respond to public health risks and PHEIC promptly and effectively within five years from the declaration of imposition of these regulations. WHO, in consultation with Member States, shall support the State Party in securing public health response capacity. The five-year period may be extended by another two years if necessary, and in exceptional circumstances, the State Party may request the WHO Director-General for a further extension, not exceeding another two years, by presenting a new implementation plan. The Director-General shall make the decision based on the technical advice of the Review Committee, and the State Party that has obtained an extension shall report to WHO on the progress.

Second, at the request of the State Party concerned, WHO shall actively collaborate with the Party by providing technical guidance and assistance, and, if necessary, mobilizing international experts for on-site assistance.

Third, if WHO declares a PHEIC in consultation with the State Party concerned, it may offer, in addition to the support mentioned above, further assistance including an assessment of the severity of the international risk and adequacy of the control measures. When conducting an on-site assessment, WHO shall mobilize international assistance and share relevant information with the State Party concerned.

Fourth, in line with the PHEIC declaration, WHO shall provide appropriate support to all State Parties that request assistance.

Of the six PHEIC declarations, the 2014 polio, 2018 Ebola, and 2020 COVID-19 declarations are ongoing. WHO regularly holds meetings of the IHR EC to determine the need to revise the recommendations and maintain the PHEIC status of these outbreaks.

As of March 25, 2020, 436,518 confirmed cases, including 19,645 deaths, of COVID-19 have been reported in 198 countries in the 6 WHO regions over a period of 80 days since China’s first official report on December 31, 2019. With the commencement of large-scale community transmission in European and Middle Eastern countries, the WHO Director-General Ghebreyesus declared COVID-19 a pandemic, based on the speed and scale of spread of COVID-19, at a briefing on March 11, 2020 (Figure 1) [3]. However, WHO has not defined the exact criteria for pandemic declaration, and so far, it has declared only influenza as a pandemic. An annual progress report, based on the Pandemic Influenza Preparedness Framework, is published to prepare for an influenza outbreak that occurs every year [4]. Although the COVID-19 pandemic declaration will not significantly change the responses of the State Parties, it has reaffirmed that the countries vulnerable to this novel infectious disease shall prepare for outbreaks more thoroughly, and WHO shall actively support and collaborate with them to accomplish the same.

As stated in the IHR, upon PHEIC declaration, WHO makes various efforts to internationally coordinate the necessary support in order to terminate the PHEIC status as soon as possible. The 2019 COVID-19 Strategic Preparedness and Response Plan was proposed by WHO on February 3, 2020 [5]. It is noteworthy that this strategy also includes accelerating the research and development (R&D) process as one of three major strategies. Since COVID-19 is a novel infectious disease identified only months ago, many aspects of the disease are yet to be discovered through research.

On February 11-12, 2020, WHO held the Global Research and Innovation Forum: Towards a Research Roadmap for COVID-19. Experts from the WHO R&D Blueprint, including myself, members of the Global Research Collaboration for Infectious Disease Preparedness, major global funders, and other experts attended the forum [6]. The fact that a COVID-19 R&D forum was convened soon after the PHEIC declaration proved the importance of R&D in the response to emerging infectious diseases. The forum selected 8 immediate research areas of priority as well as mid-term and long-term priorities in areas including epidemiology, clinical management, infection control, diagnostics, therapeutics, and vaccine development. Korea should actively participate in the COVID-19 immediate and mid/long-term priorities jointly led by WHO and its global partners to yield results that can be utilized to develop an effective global response to COVID-19.

It was a valuable experience for me to participate in the PHEIC decision process as a member of the WHO IHR COVID-19 EC. Through this experience, I learned that declaration of a PHEIC has various impacts on policy decisions depending on the country’s situation. As mentioned above, PHEIC declaration provides a rationale for WHO to actively cooperate with vulnerable countries with poor public health infrastructure and assist them in preparing for outbreaks of novel infectious diseases, such as COVID-19, and to provide technical guidance and assistance as well as mobilizing international experts for on-site assistance, if necessary. It is true that Korea is demonstrating remarkable efficiency in its response to COVID-19 by conducting high-through-put laboratory testing and even utilizing drive-through sampling centers. However, in addition to this response for early detection and screening of cases, the government should also focus on producing research-based evidence by thoroughly analyzing epidemiological, clinical and immunological data of cases, which are prerequisite for developing vaccines and therapeutics for COVID-19.

## Ethics statement

This paper is a editorial so it did not need ethical consideration.

## Figures and Tables

**Figure 1.**
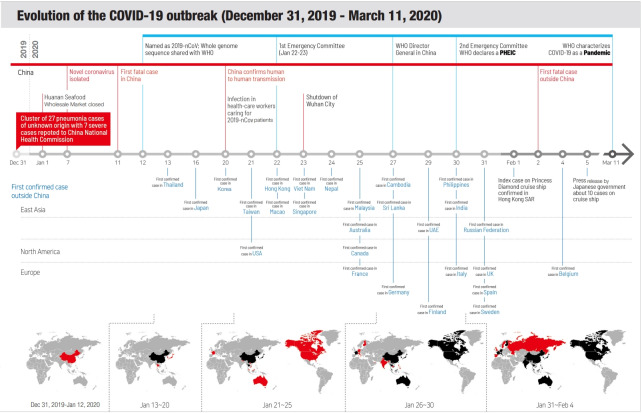
Evolution of the coronavirus disease 2019 (COVID-19) outbreak. WHO, World Health Organization; PHEIC, Public Health Emergency of International Concern. Modified from WHO. Global research and innovation forum to mobilize international action in response to the novel coronavirus (2019-nCoV) emergency [3].

## SUPPLEMENTARY MATERIALS

Korean version is available at http://www.e-epih.org/.
