# Supplementary material for: WHO International Health Regulations Emergency Committee for the COVID-19 outbreak
Source: Epidemiol Health. 2020 Mar 19;42:e2020013. doi: 10.4178/epih.e2020013 (PMC7285442; doi:10.4178/epih.e2020013)
Supplement: Supplementary file 1 [file epih-42-e2020013-suppl.docx]

**WHO 국제보건규약 COVID-19 유행 긴급위원회**

WHO International Health Regulations Emergency Committee for the COVID-19 outbreak

지영미, WHO COVID-19 긴급위원회 위원

jeey62@gmail.com

초록

2019년 12월 31일 중국 정부가 우한에서 발생한 원인불명 폐렴 사례를 공식적으로 WHO에 보고하고 2020년 1월 7일 이들 환자로부터 SARS-CoV와 유사한 신종코로나바이러스(SARS-CoV2)를 분리한 이후 3월 22일 현재 전세계적으로 거의 모든 국가에서 COVID-19 환자가 발생하였다(WHO는 2월 12일 신종코로나바이러스 감염증을 COVID-19으로 명명하였기에 이후 COVID-19이라는 공식명칭을 사용하고자 한다). WHO는 COVID-19 유행이 국제공중보건위기상황(Public Health Emergency of International Concern, PHEIC)에 해당하는 지를 논의하기 위한 국제보건규약(IHR) 긴급위원회(Emergency Committee)를 15인으로 구성하였다. 1월 22-23일과 30일 1, 2차 긴급위원회가 개최되어 PHEIC 선포 여부를 논의하였고 WHO, 중국 그리고 모든국가에 대한 권고사항을 제시하였다. 이 권고에 근거하여 한국 시간 1월 31일 새벽 WHO는 COVID-19 유행이 국제보건규약(International Health Regulations, IHR) 제12조가 규정하는 국제공중보건위기상황(Public Health Emergency of International Concern, PHEIC)임을 선포했다. WHO의 PHEIC 선포는 특히 보건 인프라가 약한 국가들이 COVID-19과 같은 신종감염병에 최대한 신속하게 대비하도록 경종을 울리는 한편 WHO가 이들 국가들에 대하여 적극적으로 지원할 수 있는 근거를 마련해준다. WHO는 2월 3일 2019 COVID-19 전략적대응대비계획(Strategic Preparedness and Response Plan, SPRP)을 발표하였으며 이 계획의 3대 분야에 연구개발 가속화를 위한 전략이 포함되었다. 이후 WHO는 2월 11-12일 COVID-19 연구개발 로드맵 작성을 위한 글로벌연구혁신포럼(Global Research and Innovation Forum: Towards a Research Roadmap)을 개최하였다. WHO가 PHEIC 선포 이후 글로벌연구혁신포럼을 개최한 것은 신종감염병 대응에서 연구개발이 얼마나 중요한 역할을 하는지를 방증한다. 한국은 COVID-19 대응에서 적극적인 실험실 검사와 drive-through 선별소 운영 등 실험실 진단에서 뛰어난 역량을 발휘하고 있는 것이 사실이나 COVID-19 유행의 역학, 임상, 면역학적인 데이터의 철저한 분석을 통해 치료제나 백신개발을 앞당기는 노력도 함께 해야 할 것이다. WHO가 글로벌 파트너들과 함께 추진 중인 COVID-19 우선순위 과제와 중장기 연구에 한국도 적극적으로 참여하여 COVID-19극복을 위한 글로벌 연구 파트너로서의 역할을 할 수 있기를 기대한다.

KEY WORDS: COVID-19, International Health Regulations, Emergency Committee, Public Health Emergency of International Concern, Research and development

2020년 1월 22/23일(1차)과 30일(2차) COVID-19 유행 대처를 위한 WHO IHR 긴급위원회(Emergency Committee)가 개최되었다. 한국 시간 1월 31일 새벽 WHO는 COVID-19 유행이 국제보건규약(International Health Regulations, IHR) 제12조가 규정하는 국제공중보건위기상황(Public Health Emergency of International Concern, PHEIC)임을 선포했다 [1]. 현재까지 PHEIC가 선포된 경우는 이번 COVID-19 유행까지 총 6번이다. 2009년 신종인플루엔자 유행을 처음으로, 2014년 야생폴리오, 2014년 서아프리카 에볼라, 2016년 지카바이러스, 2018년 에볼라 유행이 선행하는 선포 사례이다.

2005년 개정된 IHR에 의하면 각 국은 PHEIC를 선포할 만한 가능성이 있는 사태가 발생하면 24시간내에 WHO에 보고하도록 되어 있다. WHO는 각국에서 발생하는 공중보건위기 중 국제적 전파의 위험성, 여행이나 교역 제한의 위험성 등을 고려하여 긴급위원회를 구성, PHEIC 해당 여부에 대한 논의를 거쳐 WHO 사무총장이 최종 선언을 하게 된다. WHO가 국제공중보건위기상황을 선포하기 위해서는 동 규약 제2부속서(Annex 2)가 규정하는 요건을 갖추어야 한다 [1]. 그 중에는 심각한 국제적 전파의 위험성과 해당 감염병이 여행 또는 교역에 대해 미치는 영향에 대한 평가가 포함된다. 많은 감염병 유행이 매년 발생하고 WHO에 보고되는 것을 감안하면 WHO의 PHEIC 선포는 보고된 사건이 1) 공중보건에 심각한 영향을 주는가, 2) 사건이 예상치 못한 것인가, 3) 국제적 전파의 위험이 심각한가, 4) 심각한 국제교역이나 여행 제한의 위험이 있는가 등을 신중하게 평가하여 결정한다 [1].

이번 IHR COVID-19 긴급위원회는 6개 WHO 지역을 대표하는 다양한 분야 전문가 15인으로 구성되어 있다. 그들의 국적을 열거하면, 미국, 태국(2), 러시아, 프랑스, 한국, 중국, 일본, 캐나다, 네덜란드, 호주, 세네갈, 싱가폴(2), 사우디아라비아이다. 그들의 전문분야는 역학, 바이러스학 등 자연과학 외에도 커뮤니케이션도 포함된다. PHEIC 선포를 위해서는 위원들의 컨센서스가 요구되는 것이 일반적인바, 1월 22일과 23일에 개최된 1차 회의에서는 의견의 일치를 보지 못하여 PHEIC를 선포하지 못했고, 다른 나라에서 지역 내 전파가 전개되는 상황을 주시한 후인 1월 30일의 2차 회의에서야 컨센서스를 이루었다. 위원회는 확진사례가 5개의 WHO 지역에서 한 달 기간에 보고되었으며 중국 이외의 국가에서도 사람간 전파가 확인되었음에 주목했다. 위원회는 또한 국가가 사례 조기 발견, 환자 격리 및 치료, 접족자 관리 및 사회적 거리두기 등 적극적인 대응으로 바이러스 전파를 차단할 수 있다고 강조하였다. 그러나 상황이 계속 바뀌고 있 때문에 전파 예방과 방지를 위한 전략적 목표와 수단도 변경될 수 있다고 언급하였다.

WHO 사무총장은 긴급위원회의 자문, 유행국이 제공한 정보와 국제적인 확산 위험도와 여행과 교역 제한의 문제점 등을 고려하여 최종적으로 PHEIC를 선포하는 권한을 갖는다.

게브레예수스 사무총장에게 결정을 권고한 긴급위원회 성명서의 내용 중 특기할 만한 것을 간추리면 다음과 같다. (아래 3개 항목의 요약 내용은 2.3. 한국일보에 게재된 필자의 시론에 기초함 [2])

첫째, WHO가 중국에 전문가들을 파견, 합동미션을 조직해 사태에 대응한다는 것이다. 이것은 메르스 사태 때 우리나라의 전문가들과 함께 조직한 합동평가단과 유사한 형태인데, 우리나라에 대해서는 합동평가단을 먼저 가동해 본 후 국제공중보건위기상황을 선포하지 않은 데 비해, 중국에 대해서는 위기상황을 선포한 후 그러한 협력을 시행한다는 점에서 차이가 있다. 합동미션은 중국 내 신종코로나바이러스 유행과 대응 현황을 파악해 조치를 권고하도록 예정되었다. 이와 더불어, 보건의료 인프라가 취약한 나라와 지역에 대한 지원 및 그들과의 공조에 만전을 기할 것을 강조했다.

둘째, 중국이 취할 조치를 열거했다. 여기에는 WHO 및 관련 기관들에 협조할 것, 공항만에서의 출국검역을 실시하여 감염된 여행자들을 식별하라는 것이 포함되었다. 특히 출국검역을 철저히 하는 것은 중국으로부터의 여행 제한을 국제사회가 최소화하기 위한 조건이었다.

셋째, 당사국들에 대해서는 WHO가 여행과 교역에 대한 제한을 당장 권고하지 않는다는 내용이 포함되었다. 만약 사람과 물자의 국제적 이동을 제한하는 조치를 취할 때에는 공중보건을 위한 정당화 사유를 48시간 내에 WHO에 통보하고 WHO가 그러한 조치의 적절성을 판단한다는 방침을 제시했다. 아울러 당사국들은 인권존중의 원칙을 규정한 국제보건규약(IHR) 제3조에 따라, 특정 집단에 대해 낙인과 차별이 일어나지 않도록 유의해야 함을 강조했다.

WHO는 PHEIC 선포시 감염병의 국가간 전파를 최대한 막고 유행국의 대응을 지원하는 한편 유행국에 대한 과도한 교역 제한 조치가 발생하지 않게 하려는 기본적인 입장을 가지고 있다. IHR은 WHO가 PHEIC를 선포하면 다음과 같은 조치를 취할 것을 명시하고 있다(제13조 공중보건대응).

첫째, 해당국가는 공중보건위험과 국제공중보건위기상황에 신속하고 효과적으로 대응할 역량을 동 규칙 발효 시점으로부터 5년 이내에 확보하고, WHO는 회원국과 협의하여 당사국의 공중보건대응역량 확보를 지원한다. 5년의 기간은 필요시 2년 연장할 수 있으며, 예외적인 경우 당사국은 새로운 시행계획을 제시하여 2년을 초과하지 않는 범위 안에서 추가적인 기간연장을 WHO 사무총장에게 요청할 수 있다. 사무총장은 자문위원회의 의견을 고려하여 기간연장에 대한 결정을 내리며 기간을 연장 받은 해당국은 진전사항에 대하여 WHO에 보고하여야 한다.

둘째, 해당국의 요청에 따라 WHO는 기술적 지침과 지원을 제공하고 필요하면 국제전문가를 현장지원에 동원하는 등 적극적으로 협력한다.

셋째, WHO는 PHEIC를 선포하는 경우 해당국과 협의하여 위에서 언급한 지원과 함께 국제적 위험도의 심각성 및 관리조치의 적정성을 평가하는 등 해당국에 추가적 지원을 할 수 있고, 현장평가를 시행하는 경우 해당국을 위하여 국제적인 지원을 동원하며 이와 관련된 정보를 해당국과 공유한다.

넷째, WHO는 PHEIC 선포에 따라 WHO 지원을 요청하는 모든 국가에 적절한 지원을 제공하여야 한다.

이번 COVID-19까지 6회의 PHEIC 선포 중 2014년 폴리오, 2018년 에볼라와 2020년 코로나 19까지 3개가 지속되고 있다. 현재 유지되고 있는 PHEIC에 대해서 WHO는 PHEIC 선포 후 정기적으로 IHR 긴급위원회를 개최하여 권고안 수정 필요성과 PHEIC 지속 여부를 판단하게 된다.

전 세계적으로 COVID-19은 6개의 WHO 지역 189개국에서 보고되었으며, 12월 31일 중국의 공식적인 첫 보고 이후 80여일이 지나는 동안 (2020년 3월 22일 현재) 13,651명의 사망자를 포함 317,381명에 달하는 확진자가 발생하였다. 유럽과 중동지역 국가에서 지역사회 대규모 전파가 시작됨에 따라, 게브레예수스 WHO 사무총장은 3월 11일 브리핑에서 COVID-19의 전파속도와 규모에 근거하여 판데믹을 선포했다 (Figure 1) [3]. 하지만 WHO는 판데믹 선포에 대한 정확한 기준을 정하고 있지는 않으며, 현재까지는 인플루엔자에 한해서 판데믹을 선포했다. 인플루엔자에 대해서는 Pandemic Influenza Preparedness (PIP) Framework에 의거하여 매년 발생하는 유행에 대비하여 연례경과보고서를 발간한다 [4]. COVID-19 판데믹 선포 이후에 각 국의 대응이 크게 달라질 것은 없으나 신종감염병에 대한 인프라가 취약한 국가에서 앞으로의 유행에 더욱 철저히 대비하고 이를 위해 WHO가 적극적인 지원과 공조를 할 계획임을 재확인했다는 의미가 있을 것이다.

IHR에 명시된 바와 같이 WHO는 PHEIC 선포시 국제적으로 필요한 지원을 조정하고 PHEIC 상황이 최대한 빨리 종료될 수 있도록 다양한 노력을 하게 된다. 이러한 노력 중 병원체에 대한 연구개발 가속화가 포함된다. 2월 3일 WHO가 발표한 2019 COVID-19 전략적대응대비계획(Strategic Preparedness and Response Plan, SPRP)의 3대 분야에 연구개발 가속화를 위한 전략이 포함된다 [5]. COVID-19은 발견된 지 수 개월 밖에 되지 않은 새로운 감염병인 만큼 연구를 통해 밝혀내야 할 것들이 많은 상황이다.

따라서 WHO는 2월 11-12일 COVID-19 연구개발 로드맵 작성을 위한 글로벌연구혁신포럼(Global Research and Innovation Forum: Towards a Research Roadmap)을 개최하였다 [6]. 필자도 위원으로 참여하고 있는 WHO R&D 블루프린트 전문가들과 감염병대비 글로벌연구협력(GLoPID-R) 관계자, 주요 글로벌 펀더 및 기타 분야별 전문가들이 이 포럼에 참여하였다. PHEIC 선포 이후 COVID-19 연구개발 관련 포럼이 가장 먼저 개최된 것은 연구개발이 그만큼 감염병 대응에 중요하다는 점을 방증한다. 이 포럼에서는 역학, 임상, 감염관리, 진단, 치료제와 백신 개발 등 9개 분야에서 가장 우선적으로 진행되어야 할 연구와 중장기적으로 진행될 연구를 선정했다. 이후 WHO가 글로벌 파트너들과 함께 추진 중인 COVID-19 우선순위 과제와 중장기 연구에 한국도 적극적으로 참여하여 글로벌 COVID-19 대응에 활용할 수 있는 성과를 도출하는 데 앞장서야 할 것이다.

**Evolution of the COVID-19 outbreak**


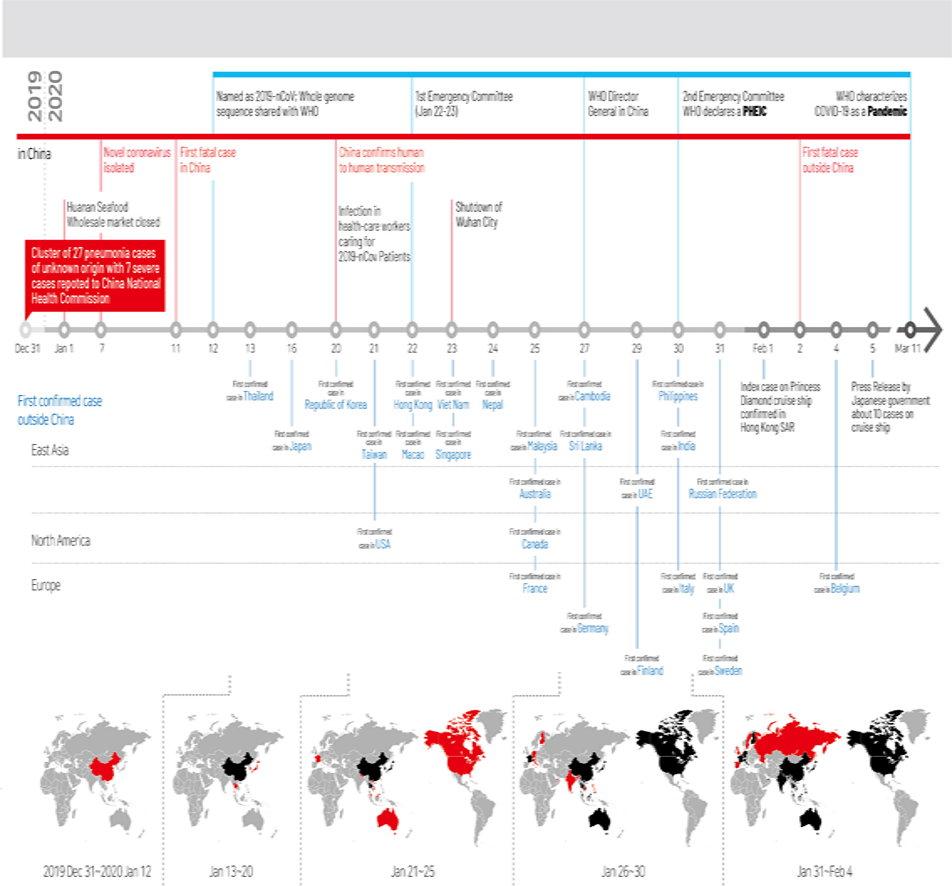


Figure 1. Evolution of the coronavirus disease 2019 (COVID-19) outbreak. WHO, World Health Organization; PHEIC, Public Health Emergency of International Concern. Modified from: WHO. Global research and innovation forum to mobilize international action in response to the novel coronavirus (2019-nCoV) emergency [3].

WHO 국제보건규약 COVID-19 긴급위원회의 일원이 되어 PHEIC 결정에 참여한 것은 의미 있는 경험이었다. 이를 통해 PHIEIC 선포가 각 국가에서 정책결정에 미치는 영향은 국가의 상황에 따라 상이함을 알 수 있었다. 위에서 언급한 바와 같이 PHEIC 선포는 보건 인프라가 약한 국가가 COVID-19과 같은 신종감염병에 대비할 수 있도록 기술적 지침과 지원을 제공하고 필요시 국제전문가를 현장지원에 동원하는 등 적극적으로 협력하기 위한 근거를 제공한다는 의미를 갖는다. 한국은 현재 high throughput 실험실 검사 능력 확보와 drive-through 선별소 운영 등 COVID-19 대응에서 세계가 부러워하는 역량을 발휘하고 있는 것이 사실이다. 그러나 이러한 방역 차원의 대응 이외에도 이번 COVID-19 유행의 역학, 임상, 면역학적인 데이터의 철저한 분석을 통해 치료제나 백신개발을 앞당기는 노력을 해야 할 것이다.

참고문헌

1. World Health Organization. International health regulations (2005). 3rd ed; 2016 [cited 2020 Feb 12]. Available from: https:// www.who.int/ihr/publications/9789241580496/en/.

2. Jee Y. PHEIC declared: what does WHO say?; 2020 Feb 3 [cited 2020 Feb 10]. Available from: https://www.hankookilbo.com/ News/Read/202002021589772383 (Korean).

3. World Health Organization. Global research and innovation fo­rum to mobilize international action in response to the novel cor­onavirus (2019-nCoV) emergency; 2020 Feb 11-12 [cited 2020 Mar 22]. Available from: https://www.who.int/news-room/events/ detail/2020/02/11/default-calendar/global-research-and-innova­tion-forum-to-mobilize-international-action-in-response-to-the-novel-coronavirus-(2019-ncov)-emergency.

4. World Health Organization. Pandemic influenza preparedness (PIP) framework [cited 2020 Mar 20]. Available from: https:// www.who.int/influenza/pip/en/.

5. World Health Organization. 2019 Novel coronavirus (2019‑nCoV): strategic preparedness and response plan: 2020 Feb 3 [cited 2020 Mar 22]. Available from: https://www.who.int/docs/default-source/ coronaviruse/srp-04022020.pdf.

6. World Health Organization. A research and development Blue­print for action to prevent epidemics [cited 2020 Mar 22] Availa­ble from: https://www.who.int/blueprint/en/.
